# Enhancement of the Immunogenicity and Protective Efficacy of a Mucosal Influenza Subunit Vaccine by the Saponin Adjuvant GPI-0100

**DOI:** 10.1371/journal.pone.0052135

**Published:** 2012-12-17

**Authors:** Heng Liu, Harshad P. Patil, Jacqueline de Vries-Idema, Jan Wilschut, Anke Huckriede

**Affiliations:** Department of Medical Microbiology, Molecular Virology Section, University of Groningen, University Medical Center Groningen, Groningen, The Netherlands; University of Pittsburgh, United States of America

## Abstract

Identification of safe and effective adjuvants remains an urgent need for the development of inactivated influenza vaccines for mucosal administration. Here, we used a murine challenge model to evaluate the adjuvant activity of GPI-0100, a saponin-derived adjuvant, on influenza subunit vaccine administered via the intranasal or the intrapulmonary route. Balb/c mice were immunized with 1 µg A/PR/8 (H1N1) subunit antigen alone or in combination with varying doses of GPI-0100. The addition of GPI-0100 was required for induction of mucosal and systemic antibody responses to intranasally administered influenza vaccine and significantly enhanced the immunogenicity of vaccine administered via the intrapulmonary route. Remarkably, GPI-0100-adjuvanted influenza vaccine given at a low dose of 2×1 µg either in the nares or directly into the lungs provided complete protection against homologous influenza virus infection.

## Introduction

As the respiratory tract is the portal of entry for influenza virus, it has long been an issue to develop mucosal vaccines to elicit influenza-specific immunity at the site for disease prevention. Successful mucosal immunization is supposed to elicit high titers of secretory IgA (SIgA) that can neutralize extracellular viruses at the luminal site of the respiratory epithelium, or intracellular viruses during transcytosis. [1] Together with innate immunity, SIgA provides a first line of host defence against virus infection.[1]–[3] In addition, mucosal immunization can imprint activated lymphoctyes with surface markers that will preferentially direct them to home to mucosal sites. These lymphocytes can be quickly re-activated upon virus infection and can contribute to efficient viral clearance. Apart from immunological benefits, mucosal immunization has several important advantages over parenteral immunization. [4], [5] Mucosal immunization prevents the potential safety risk caused by contaminated needles, spares time and cost involved in parenteral vaccine administration by health care workers and improves vaccination acceptance by the general population.

So far, the only marketed influenza vaccine for mucosal administration is live attenuated influenza vaccine (LAIV) delivered as large droplet aerosol via the intranasal route. [4], [6] LAIV contains recombinant viruses composed of a viral backbone of a cold-adapted virus strain with two RNA segments encoding hemagglutinin (HA) and neuraminidase (NA) from circulating strains. Many studies have shown that LAIV is effective in inducing both systemic and mucosal immunity with a better cross-protective efficacy against heterologous virus strains, which persists for a longer time span compared to immunity by parenterally administered inactivated virus vaccines.[4], [7]–[9] Nevertheless, young children and the elderly, the vulnerable populations who are among the major targets for influenza vaccination programs, are excluded from the application of LAIV due to their weak immune systems and the potential risk of disease development. Moreover, there has been a concern about the emergence of virulent virus strains from the vaccine virus strain by genetic mutation or re-association with wild-type virus strains.

Mucosal vaccines containing inactivated virus or isolated viral proteins are preferable from a safety point of view. However, such formulations possess relatively weak immunogenicity.[4], [10]–[12] Accordingly, mucosal adjuvants are required to break down the immune-tolerant nature of the mucosal environment and to stimulate vaccine immunogenicity. Bacterial enterotoxins such as cholera toxin from *Vibrio cholera* and heat-labile enterotoxin from *Escherichia coli* have long been known to possess strong mucosal adjuvant activity. [5], [13] However, the associated toxicity and the induced side-effects have prohibited their use in human vaccines and even led to retraction of an already marketed nasal influenza vaccine. [14], [15] Development of safe novel adjuvants with strong immune-potentiating capacity but with acceptable reactogenicity therefore remains an urgent need for mucosal vaccine research.

GPI-0100 is a semi-synthetic triterpenoid glycoside. It is derived from QS-7, one of the purified components of Quil A, a saponin adjuvant extracted from the bark of the Molina tree *Quillaia saponaria*.[16]–[18] GPI-0100 shows a better safety profile and increased stability in aqueous solution at physiological pH when compared with other saponin-derived adjuvants. GPI-0100 has been used in clinical trials for cancer vaccines. Specifically, a study on a candidate prostate cancer vaccine indicates that there are no serious side effects at an adjuvant dose of up to 3000 µg. [19] In an earlier study, we showed that GPI-0100 significantly enhances both humoral and cellular immune responses elicited by influenza subunit vaccine when delivered intramuscularly. [20] The enhancement was observed in both the Th1 (IgG2a and IFN-γ) and the Th2 (IgG1 and IL-4) arm of the immune response. Remarkably, the adjuvanted vaccine induced significant protection against influenza virus growth in the lung even at an extremely low antigen dose (0.04 µg HA). In contrast, in the absence of adjuvant a 25-fold higher antigen dose (1 µg HA) was required to achieve the same level of lung protection. Aside from its systemic adjuvant activity, GPI-0100 also showed mucosal adjuvant activity in a vaccine for *Porphyromonas gingivalis*. [21] When applied via the intranasal route, GPI-0100 strongly potentiated both systemic and mucosal antibody responses specific for the antigen hemagglutinin B (HagB) of this bacterium.

Here, we evaluated the mucosal adjuvant activity of GPI-0100 in conjunction with A/PR/8 (H1N1) influenza subunit vaccine in mice. We compared the capacity of non-adjuvanted and GPI-0100-adjuvanted influenza vaccine delivered via the upper (nose) or the lower (lung) respiratory tract to induce mucosal and systemic immune responses and protection from virus challenge. We show that induction of systemic and mucosal immune responses by intranasal vaccine requires adjuvantation with GPI-0100. Intrapulmonary vaccines induced local and systemic antibody responses even without adjuvantation but these responses were significantly increased upon GPI-0100 adjuvantation. Moreover, complete inhibition of virus growth in the lungs was achieved only by the adjuvanted vaccines for both administration routes. We therefore consider GPI-0100 a potential candidate adjuvant for mucosal influenza vaccines.

## Materials and Methods

### GPI-0100

GPI-0100 was purchased from Hawaii Biotech, Inc. (Aiea, HI, USA) as powder and was stored at 4°C. A stock solution of GPI-0100 (10 mg/ml) was prepared in HBS buffer (5 mM Hepes, 150 mM NaCl and 0.1 mM EDTA, pH 7.4). After centrifugation through a Spin-X centrifuge tube filter (Corning, MA, USA), the sterile stock solution was stored at 4°C for use within one month.

### Virus and Subunit Vaccine

A stock of A/PR/8 (H1N1) influenza virus propagated on Madin–Darby canine kidney (MDCK) cells was kindly provided by Solvay Biologicals (Weesp, Netherlands). The virus was further propagated on embryonated chicken eggs by us and the virus titer was determined by measuring the tissue culture infectious dose 50 (TCID_50_). To this end, serial 2-fold dilutions of virus suspension were inoculated on MDCK cells grown in serum-free medium. 1 hr later TPCK trypsin (Sigma, Zwijndrecht, Netherlands) was added to a final concentration of 5 ng/ml. After 72 hr, supernatants were collected and transferred to a V-bottom 96-well plate followed by the addition of 50 µl 1% guinea pig erythrocytes (Harlan, Horst, Netherlands) to each well. The plate was incubated for 2 hr at room temperature before reading. The titer was determined as the highest virus dilution at which hemagglutination was visible and the TCID_50_ was calculated by the method of Reed & Muench. [20].

For virus inactivation, the virus was incubated with freshly diluted β-propiolactone in citrate buffer (125 mM sodium citrate, 150 mM sodium chloride, pH 8.2) at a final concentration of 0.1% β-propiolactone. The inactivation procedure was carried out for 24 hr at 4°C under continuous stirring. After inactivation, the virus was dialyzed against HBS buffer overnight at 4°C. Subunit vaccine was prepared by solubilizing the inactivated virus (0.8 mg virus protein/ml) in HBS buffer containing Tween 80 (0.6 mg/ml) and hexadecyltrimethylammonium bromide (CTAB, 3.0 mg/ml) for 3 hr at 4°C under continuous stirring, followed by the removal of viral nucleocapsid from the preparation by ultracentrifugation for 30 min at 50,000 rpm in a TLA100.3 rotor at 4°C. Detergents were then removed by overnight absorption onto Biobeads SM2 (634 mg/ml, Bio-Rad, Hercules, Canada) washed with methanol prior to use.

Protein content of the inactivated virus and subunit material was determined by a modified Lowry assay. [22] Hemagglutinin (HA) content was assumed to be equal to the total protein for subunit material based on sodium dodecyl sulfate polyacrylamide gel electrophoresis (SDS-PAGE) results. [23] Vaccines were mixed at the indicated amounts of subunit and GPI-0100 just before immunization.

### Animal Handling

The protocol for the animal experiments described here was approved by the Ethics Committee on Animal Research of the University of Groningen (Permit number: DEC 5896D).

For immunization experiments, female Balb/c mice (Harlan) aged 8–10 weeks were grouped (n  = 6 per group) and immunized via the intramuscular, intranasal or intrapulmonary route with 1 µg A/PR/8 subunit vaccine with or without GPI-0100 adjuvant in a two-dose immunization regimen (day 0 and day 20). For intramuscular immunization, vaccines in 50 µl were divided over both hind legs. For intranasal immunization, vaccines in 5 µl were given by pipet to both nasal nares slowly. For intrapulmonary immunization, mice were brought to an upright position after isoflurane anesthesia and the trachea was intubated with a modified Autogard catheter (Becton Dickinson BV, Breda, Netherlands). Vaccines in 50 µl were then administered using a IA-1C Microsprayer Aerosolizer for mice attached to a FMJ-250 High Pressure Syringe (both from Penn-Century Inc., PA, USA). Control mice were intramuscularly injected with HBS buffer. Pre-boost serum samples were collected on day 20 by orbital puncture prior to immunizations. On day 27, mice were sacrificed and nose wash, lung wash, serum and spleen samples were collected for ex vivo immuno-assays. Mucosal wash samples were collected in 1 ml phosphate buffer saline (PBS) containing protease inhibitor (Complete Protease Inhibitor Cocktail, Roche, IN, USA).

For challenge experiments, mice received the immunization regimen as described above. Pre-boost and pre-challenge serum samples were collected on day 20 and 34 by orbital puncture prior to immunizations or virus infections respectively. On day 34, mice were infected intranasally with 200 TCID_50_ A/PR/8 influenza virus in 50 µl of HBS buffer. The virus infection was carried out under isoflurane anesthesia to ensure deposition of the virus into the lungs. Mice were monitored, twice a day at fixed time points, for clinical signs of illness including weight loss and changes in behavior or appearance. Mice were bled and sacrificed on day 37. Nasal wash, serum and spleen samples were collected for immuno-assays. The lung lobes were collected in 1 ml PBS for homogenization and the processed samples were stored at −80°C for later determination of lung virus titers.

To evaluate the safety of mucosal administration of GPI-0100, mice were grouped (n  = 3 per group) and received two doses of GPI-0100 alone via the intranasal or the intrapulmonary route. For intranasal delivery, GPI-0100 in 10 µl was given by pipet to both nasal nares slowly. For intrapulmonary immunization, GPI-0100 in 50 µl was given as described above. The control mice for both routes received HBS buffer. Lung samples were collected 72 hr after the second administration for histopathological analysis.

### IgA, IgG, IgG1 and IgG2a ELISAs

Influenza HA-specific antibody responses were determined by ELISA. [23] Briefly, ELISA plates (Greiner, Alphen a/d Rijn, Netherlands) were coated with 0.2 µg of PR8 influenza subunit antigen per well. 2-fold serial dilutions of serum samples in PBST (0.05% Tween 20 in PBS) were applied to the wells in duplicate and incubated for 1.5 hr. Horseradish peroxidase-conjugated goat anti-mouse IgA or IgG (SouthernBiotech, AL, USA) was added and incubated for 1 hr for the detection of H1N1-specific IgA, IgG, IgG1 or IgG2a antibodies. All incubations were carried out at 37°C. The staining was performed with substrate buffer (50 mM citrate-phosphate buffer, pH 5.5, containing 0.04% o-phenylenediamine and 0.012% H_2_O_2_). Absorbance at 492 nm (O.D.492) was measured using an ELISA reader (Bio-tek Instruments Inc., VT, USA). For IgA response, the average OD at 492 nm (OD492, with the standard error of the means (S.E.M.)) for each group at each dilution was calculated. For IgG response, the averaged titer for each group (with the S.E.M.) was calculated as the (10log of the) reciprocal of the sample dilution corresponding to an OD492 of 0.2. For calculation purposes, sera with titers below the detection limit were assigned an arbitrary titer corresponding to half of the detection limit.

Calibration plates for IgG1 and IgG2a assay were coated with 0.1 µg goat anti-mouse IgG (SouthernBiotech). Increasing concentrations of purified mouse IgG1 or IgG2a (SouthernBiotech) were added to the plates. Average IgG1 and IgG2a responses for each group are given as concentrations (µg/ml) of influenza HA-specific IgG1 and IgG2a.

### Hemagglutination Inhibition (HAI) Assay

Serum samples were pre-heated at 56°C for 30 min to inactivate serum proteins. [23] After cooling down, 75 µl of the processed serum samples were treated with 225 µl of Kaolin for 30 min at room temperature followed by centrifugation at 1500 rpm for 10 min. The supernatant was collected and applied to a V-bottom 96-well plate for 2-fold serial dilutions. The same volume of PR8 influenza virus dilution containing 4 HAU virus was added to each well and allowed an incubation for 40 min at room temperature. 50 µl of 1% guinea pig erythrocytes were then added to each well and the plate was incubated for another 2 hr before reading. The titer was determined as the highest serum dilution at which hemagglutination inhibition was visible. 2log HAI titer for individual mouse is presented.

### Elispot

ELISA plates were coated with purified rat IgG1 against mouse IFN-γ or IL-4 (BD Pharmingen, CA, USA). [23] Freshly isolated splenocytes (500,000 cells per well) were added to the plates in triplicate in medium containing 5% fetal calf serum with or without PR8 subunit (1 µg per well). After an overnight incubation at 37°C, cells were lysed in ice-cold water and plates were washed. IFN-γ or IL-4 detection was carried out by 1 hr incubation with biotinylated anti-mouse IFN-γ or IL-4 antibody followed by subsequent incubation with streptavidin-alkaline phosphatase (Pharmingen) for 1 hr. Spots were developed by adding 100 µl of substrate solution to each well. The substrate solution included 5-bromo-4-chloro-3-indolylphosphate in water containing 6 mg/ml agarose (Sigma), 9.2 mg/ml 2-amino-2-methyl-1-propanol (Sigma) and 0.08 µl/ml Triton X-405 at 1 mg/ml. The plates were further incubated for 3 hr at 37°C. Images of the plates were taken by an automated ELI-spot assay video analysis system (A EL VIS, Hannover, Germany). Spots were counted manually. Spots observed in the wells without PR8 subunit (backgrounds) were subtracted from the spots observed in the stimulated wells. Number of influenza-specific IL4-secreting cells per 500,000 splenocytes for each mouse is given.

### Determination of Virus Titer in Lungs of the Challenged Mice

Lungs collected from the immunized and challenged mice were homogenized and stored at −80°C until use. [23] Virus titers were determined by inoculating serial dilutions of the clarified homogenates on MDCK cells and culturing the cells in the presence of TPCK-trypsin as described earlier. The highest dilution that still resulted in hemagglutination was taken as the virus titer in the lungs. Result from individual mouse is presented as 10log virus titer per gram of lung tissue.

### Lung Histology

Lung samples from the safety evaluation experiment were harvested for histopathological analysis. Briefly, lungs were inflated by injection of 1 ml Tissue-Tek OCT compound (Sakura, Alphen aan den Rijn, Netherlands) through the trachea and snap frozen in liquid nitrogen. The lung samples were then stored at −80°C until use. Frozen sections of lungs (5 µm thick) were prepared using a Leica CM 1950 micotome (LEICA, Rijswijkn, Netherlands) and stored at −80°C until use. Just prior to staining, the sections were fixed in 100% acetone for 10 minutes and air-dried at room temperature for 30 minutes. Tissues were rehydrated with PBS and blocked for endogenous peroxidase with H_2_O_2_. Sections were washed three times with PBS and stained for neutrophils and macrophages using rat anti mouse Ly-6G and Ly-6C (BD pharmingen) and rat anti mouse CD68 (BD pharmingen) respectively for 60 minutes. Sections were washed as mentioned previously and subsequently incubated with horseradish peroxidase-conjugated goat anti-rat antibody for 30 minutes. After washing, color was developed using an AEC staining kit (Sigma). Sections were washed again and stained with Mayer’s Haematoxylin for 10 minutes. Slides were washed with tap water for 5–10 minutes and mounted with ImmunoHistomount. The processed slides were then scanned with a NanoZoomer 2.0-HT (Hamamatsu, Tohoku, Japan) and the scanned images were analyzed by HistoQuest software (TissueGnostics, Vienna, Austria). The results are presented as % neutrophils or % macrophages per stained sample.

### Statistical Analysis

The unpaired Student’s t-test was used to determine if the differences in influenza-specific responses observed between groups of mice were significant. A p value of p<0.05 was considered significant. Spearman (nonparametric) correlation analysis was performed to show the inverse relationship observed between influenza-specific IgG responses and lung virus titers.

## Results

### Effect of GPI-0100 on Lung Protection Induced by Mucosally Administered Influenza Vaccine

**Figure 1:**
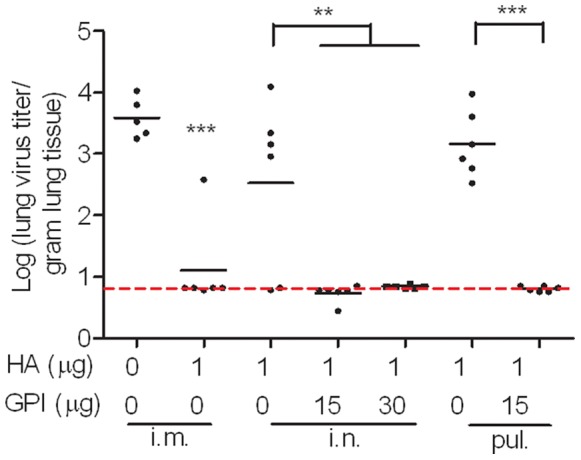
Effects of unadjuvanted and GPI-0100-adjuvanted mucosal influenza vaccine on lung virus titers. Mice were immunized on day 0 and on day 20 with 1 µg A/PR/8 subunit vaccine alone or adjuvanted with the indicated doses of GPI-0100. The control mice received HBS buffer. The immunizations were given via intramuscular (i.m.), intranasal (i.n.) or intrapulmonary (pul.) route. 2 weeks after the second immunization, mice were infected with live A/PR/8 virus. Lung samples were collected 3 days later upon termination. Virus titer is expressed as the 10log virus titer per gram of lung tissue for individual mice. The black line represents the geometric mean virus titer per group. One mouse from the HBS control group was sacrificed before the challenge due to abnormal tissue growth with unknown reason. Levels of significance are depicted as follows: *p<0.05, **p<0.01 and ***p<0.005.

To evaluate the protective efficacy of non-adjuvanted and GPI-0100-adjuvanted influenza vaccine administered in the nose or in the lungs, mice were immunized twice with a 20-day interval and were infected with live A/PR/8 virus 2 weeks after the second immunization. No significant weight loss was observed after the virus challenge in any of the experimental groups for three days till sacrifice. On day 3, generally the day at which lung virus titers are at maximum, we sacrificed the mice, collected lung samples, and determined virus titers in the lung homogenates. Absence of detectable virus in the lung or significant reduction as compared to control mice at this time point is generally regarded as indication for protection.[24]–[26] The HBS buffer group showed an average lung virus titer of 3.6-logs (Figure 1). Lungs of mice that received plain influenza vaccine via the intramuscular route were significantly protected against virus growth; 5 out of 6 lung samples collected from these mice had virus titers below the detection limit. On the other hand, mice that received plain influenza vaccine via the intranasal and or the intrapulmonary route developed average lung virus titers not statistically different from those in the buffer control group. In contrast, mice immunized with GPI-0100-adjuvanted influenza vaccine via either of the mucosal routes showed complete protection against lung virus growth, none of the lung samples collected from these mice had detectable lung virus titers. These data indicate that GPI-0100 is a strong mucosal adjuvant for influenza vaccine.

### Effect of GPI-0100 on Serum Antibody Responses Elicited by Mucosal Influenza Vaccine

**Figure 2:**
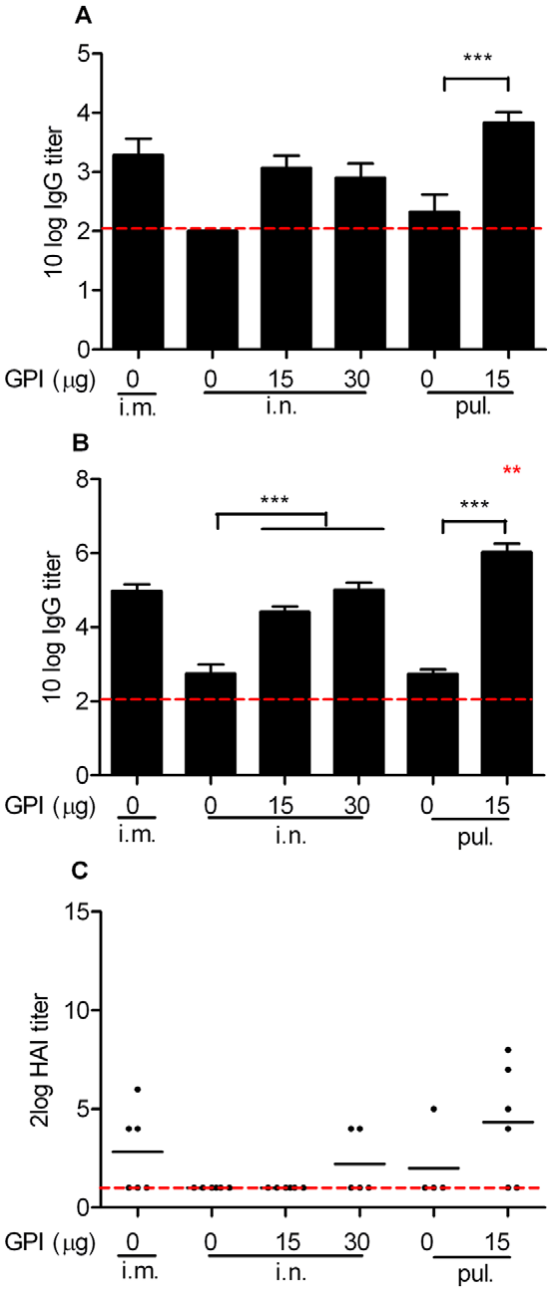
Influenza-specific IgG and hemagluttination inhibition (HAI) titers elicited by unadjuvanted and GPI-0100-adjuvanted mucosal influenza vaccine. Serum samples from the mice described in the legend to Fig. 1 were collected on day 20 and day 34. (A) Total IgG responses after a single immunization. Average 10log IgG titers ± standard error of the mean (S.E.M.), n  = 6 mice per group. The detection limit of the assay is represented by the dotted line. (B) Total IgG responses after two immunizations. (C) HAI titers after two immunizations. The results are expressed as 2log HAI titers of individual mice. The black line represents the geometric mean virus titer per group. Due to technical reasons only 5 and 4 samples from mice receiving 30 µg GPI-0100 adjuvanted intranasal immunization and unadjuvanted intrapulmonary immunization were available for the HAI assay.

Systemic humoral immune responses elicited by mucosal influenza subunit vaccine were evaluated by performing influenza-specific IgG ELISAs on serum samples collected on day 20 and day 34 from mice immunized in Figure 1. Serum samples collected 20 days after a single immunization showed that plain influenza vaccine could effectively induce IgG responses when administered via the intramuscular route (Figure 2A). None of the mice that received plain influenza vaccine via the intranasal route and only 2 out of the 6 pulmonarily vaccinated mice developed detectable IgG responses after one immunization. The responses were significantly enhanced upon GPI-0100 adjuvantation; 11 out of 12 mice that received the adjuvanted intranasal vaccine developed detectable IgG titers after a single immunization and all mice receiving the adjuvanted intrapulmonary vaccine developed IgG titers 3-logs after a single immunization.

Influenza-specific systemic antibody responses were further enhanced after the second immunization, as shown by antibody determination in serum samples collected prior to the virus challenge (Figure 2B). Again, strong GPI-0100 adjuvant effects were observed for both intranasal and intrapulmonary influenza vaccine (p<0.0001 for the comparison between adjuvanted and plain intranasal vaccine; p = 0.0006 for the comparison between adjuvanted and plain intrapulmonary vaccine). Moreover, booster immunizations with adjuvanted vaccine were very effective (increase of serum IgG titers by a factor of 130–220), while booster immunization with plain vaccine increased IgG titers only moderately (factor 50). Mice that received the adjuvanted influenza vaccine via the intranasal route all developed serum IgG titers similar to those found in mice immunized by standard intramuscular injection. Notably, the group that received the adjuvanted vaccine via the intrapulmonary route developed the highest serum IgG titers among all the treatment groups (p = 0.0023 for the comparison between plain intramuscular and adjuvanted intrapulmonary vaccine; p = 0.0032 for the comparison between adjuvanted intranasal and intrapulmonary vaccine).

We further evaluated the hemagglutination inhibition (HAI) capacity of the pre-challenge serum samples mentioned above. HAI titers to PR8 virus are generally low or undetectable, possibly due to the intrinsically low immunogenicity of this virus strain. 3 out of the 6 mice receiving plain influenza vaccine intramuscularly developed detectable HAI titers with an average titer of 7 (Figure 2C). However, only 1 out of the 10 mice receiving plain vaccine via any of the mucosal routes developed detectable HAI titers. Upon GPI-0100 adjuvantation, a higher number of mucosally immunized mice developed positive HAI titers with the highest responses observed in mice immunized via the intrapulmonary route. Interestingly, to induce a positive HAI titer, intranasal influenza vaccine required a higher GPI-0100 dose than intrapulmonary influenza vaccine. Together, these data demonstrate that use of GPI-0100 as adjuvant in mucosal influenza vaccines significantly stimulates systemic humoral immune responses. The observed systemic antibody responses correlated strongly with protection from virus growth in the lungs.

### Effect of GPI-0100 on the Serum IgG Subtype and Th Cell Profile Induced by Mucosal Influenza Vaccine

**Figure 3:**
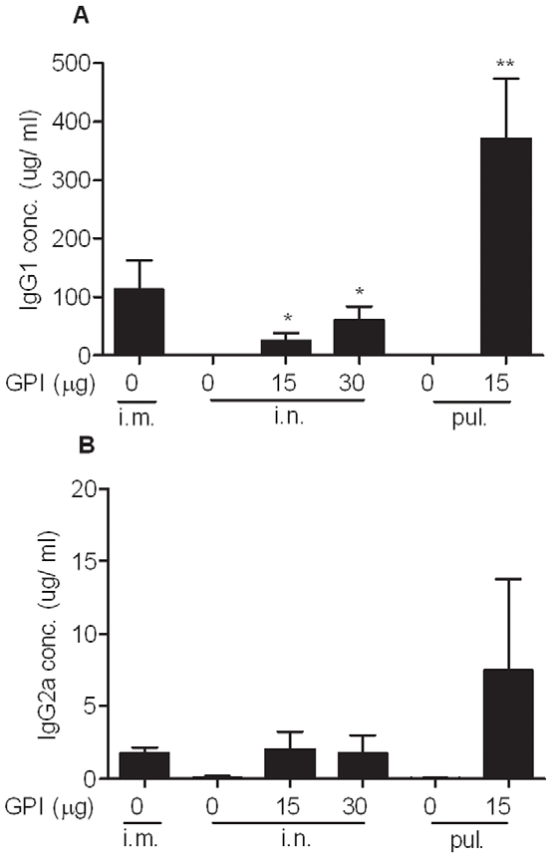
Phenotype of the influenza-specific antibody responses. Serum samples from the mice described in the legend to Fig. 1 were collected on day 37. (A) Average quantities (µg/ml) of influenza-specific IgG1± S.E.M., *n*  = 6 mice per group. (B) Average quantities (µg/ml) of influenza-specific IgG2a ± S.E.M.

To characterize the phenotype of the systemic antibody responses, we performed influenza-specific IgG1 and IgG2a ELISAs on the post-challenge serum samples collected on day 37 upon sacrifice. Mice that received plain influenza vaccine via the intramuscular route developed a Th2-skewed antibody response with mainly IgG1 production (Figure 3). Mice receiving the same vaccine via either one of the mucosal routes, on the other hand, failed to develop detectable IgG1 or IgG2a responses. The induction of both antibody subtypes elicited by mucosal influenza vaccine was substantially enhanced upon GPI-0100 adjuvantation. Yet, the humoral immune responses elicited by the adjuvanted mucosal influenza vaccine remained Th2 skewed.

**Figure 4:**
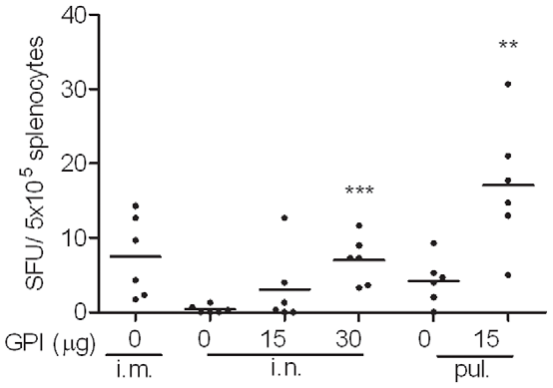
Effect of GPI-0100 on influenza-specific IL-4-producing T cells. Mice were immunized on day 0 and on day 20 with 1 µg A/PR/8 subunit vaccine alone or adjuvanted with the indicated doses of GPI-0100. The immunizations were given via intramuscular (i.m.), intranasal (i.n.) or intrapulmonary (pul.) route. Spleen samples were collected one week later upon termination. Splenocytes were isolated and stimulated overnight with PR8 subunit. The result is expressed as cytokine producing splenocytes per 5×10^5^ cells of individual mice.

To evaluate the cellular immune response elicited by mucosal influenza vaccine, mice were immunized twice with a 20 day interval and were sacrificed one week after the second immunization. Elispot assays performed on the collected splenocytes showed that all of the tested vaccines failed to induce detectable numbers of IFNγ-producing T cells (data not shown). IL-4 Elispot assay revealed that plain influenza vaccine administered via the intramuscular route elicited Th2 cellular immune responses (Figure 4). Intranasal immunization, however, was inefficient in eliciting IL-4-secreting T cells in the immunized mice unless GPI-0100-adjuvanted vaccine was used. Intrapulmonary influenza vaccine was capable of inducing IL-4-secreting T cells in the absence and presence of GPI-100 but the number of cells was significantly enhanced upon adjuvantation (p = 0.0033). The cellular immune responses observed here showed a strong relation to serum IgG antibody titers. Moreover, the Th2 type cellular immune response elicited by the tested vaccines was consistent with the phenotype of the serum antibody responses.

### Effect of GPI-0100 on Mucosal Antibody Responses Elicited by Mucosal Influenza Vaccine

**Figure 5:**
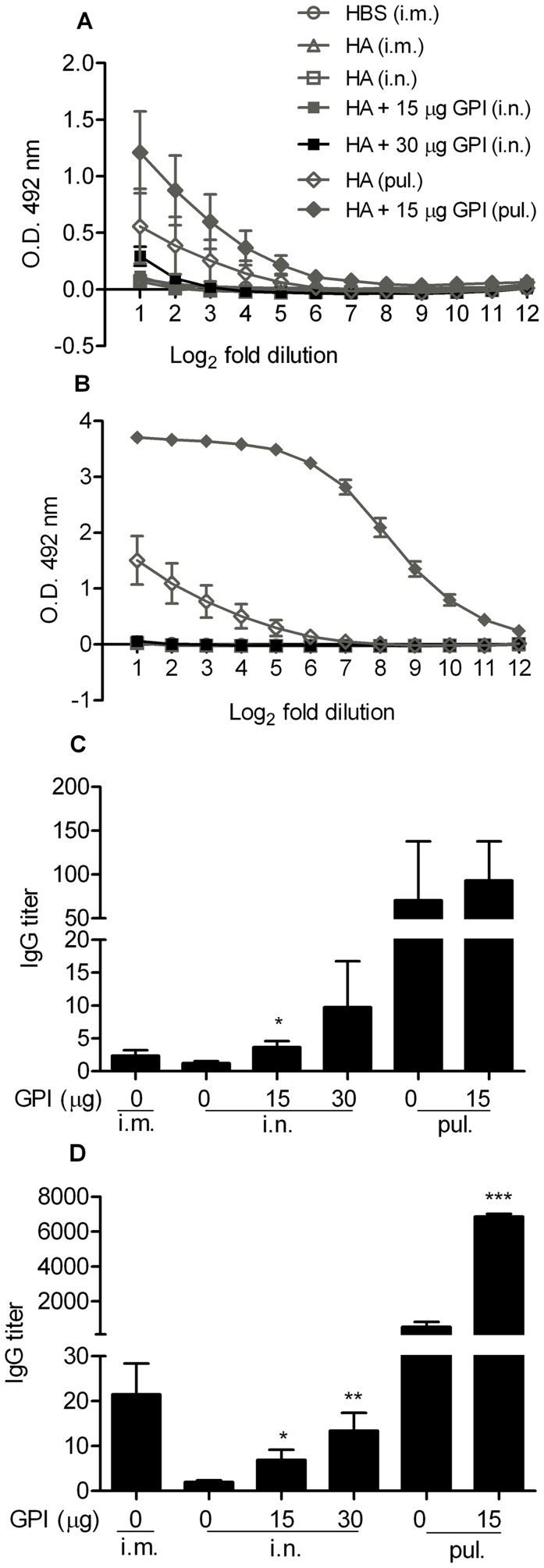
Influenza-specific mucosal IgA and IgG responses elicited by unadjuvanted and GPI-0100-adjuvanted mucosal influenza vaccine. Nose and lung wash samples from the mice described in the legend to Fig. 4 were collected on day 27 upon termination. (A) Nasal IgA responses after two immunizations. Average OD492 at each dilution ± S.E.M., n  = 6 mice per group. The starting and ending dilution is 2 and 4096 respectively. (B) Lung IgA responses after two immunizations. (C) Nasal IgG responses after two immunizations. Average IgG titers ± S.E.M. Due to technical reasons only 5 samples from mice receiving unadjuvanted intrapulmonary immunization were available. (D) Lung IgG responses after two immunizations. Due to technical reasons only 5 samples from mice receiving HBS and intrapulmonary immunization were available.

To evaluate SIgA antibody responses elicited by mucosal influenza vaccine, nasal and lung wash samples were collected from the mice described above for the evaluation of cellular immune response. Influenza-specific IgA ELISA performed on the nasal wash samples showed that plain influenza vaccine did not induce detectable SIgA responses in mice when administered via the intramuscular or the intranasal route (Figure 5A). Upon high dose GPI-0100 adjuvantation, intranasal influenza vaccine induced detectable nasal SIgA responses in 3 out of the 6 immunized mice. Interestingly, when administered via the intrapulmonary route even plain influenza vaccine induced a substantial nasal SIgA response. This response was further enhanced by adjuvantation with a low dose of GPI-0100. Influenza-specific IgA ELISA performed on the lung wash samples showed that only mice receiving intrapulmonary immunization developed detectable lung IgA responses (Figure 5B). Moreover, the responses were significantly enhanced upon GPI-0100 adjuvantation (p = 0.0001).

Since the presence of mucosal IgG antibody has also been suggested to play a role in lung protection, we evaluated influenza-specific IgG in the collected mucosal wash samples described above. [3], [27], [28] IgG titers were barely detectable in nasal wash samples collected from mice receiving plain influenza vaccine via the intramuscular or the intranasal route (Figure 5C). GPI-0100-adjuvanted intranasal influenza vaccine, however, did induce detectable nasal IgG titers in most of the immunized mice. Intrapulmonary delivery of non-adjuvanted vaccine resulted in detectable nasal IgG titers in 3 out of 5 mice. When receiving GPI-0100 adjuvanted intrapulmonary vaccine all mice responded and the nasal IgG titers were generally higher than those for the non-adjuvanted vaccine. IgG ELISA performed on lung wash samples revealed that all mice immunized with plain influenza vaccine via the intramuscular route developed detectable IgG titers in the lungs (Figure 5D). The IgG antibody we observed here probably was derived from systemic IgG that transudated to the lungs rather than from locally produced IgG. As observed earlier in nasal wash samples, substantial IgG titers could only be detected in lung wash samples collected from intranasally immunized mice that received GPI-0100 adjuvanted vaccine. Notably, mice receiving plain influenza vaccine via the intrapulmonary route developed quite high lung IgG titers. The titers were further enhanced by GPI-0100 (p<0.0001). Thus, GPI-0100 significantly stimulates mucosal antibody responses elicited by mucosal influenza vaccine.

### Effect of GPI-0100 on Lung Inflammation

**Figure 6:**
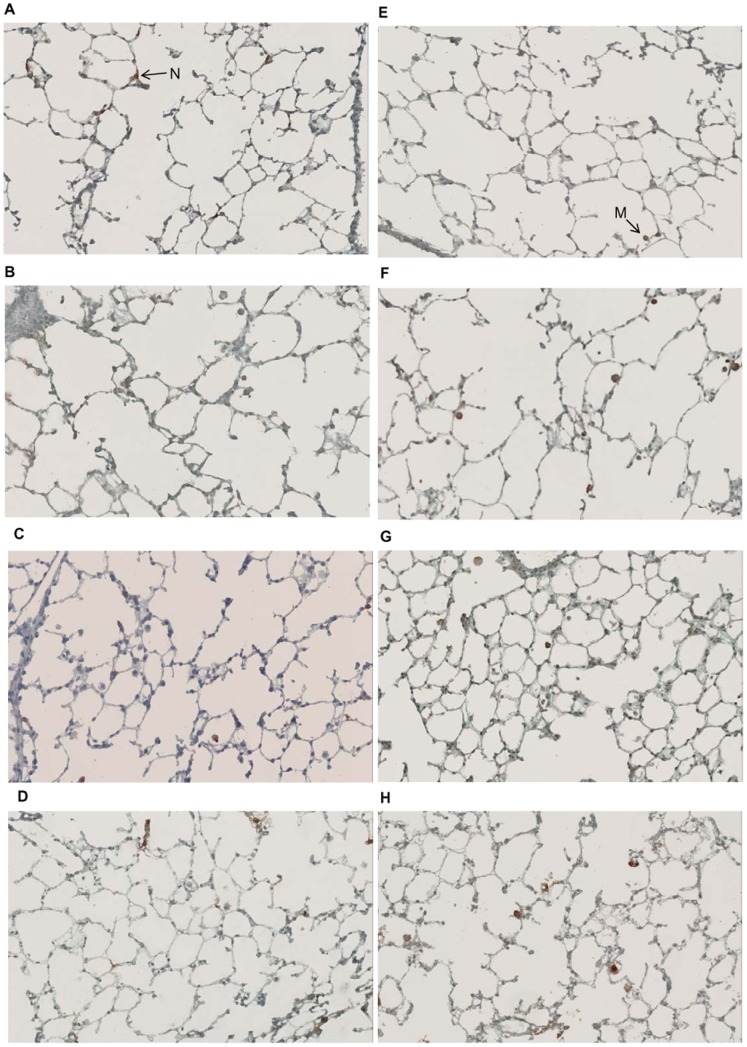
Effect of GPI-0100 on lung histology. On day 0 and on day 20 mice received either HBS buffer (A, C, E, G) or GPI-0100 at the indicated doses (B, D, F, H) via the intranasal (A, B, E, F) or the intrapulmonary (C, D, G, H) route. Lung samples were collected 3 days after the second treatment upon termination. Lung sections were prepared and stained for Ly-6G and Ly-6C (A-D) or CD68 (E-H) to identify neutrophils and macrophages, respectively. Representative pictures of histological analyses of each treatment group are shown. The brown colored cells indicated by an arrow is a neutrophil (N) or a macrophage (M).

**Figure 7:**
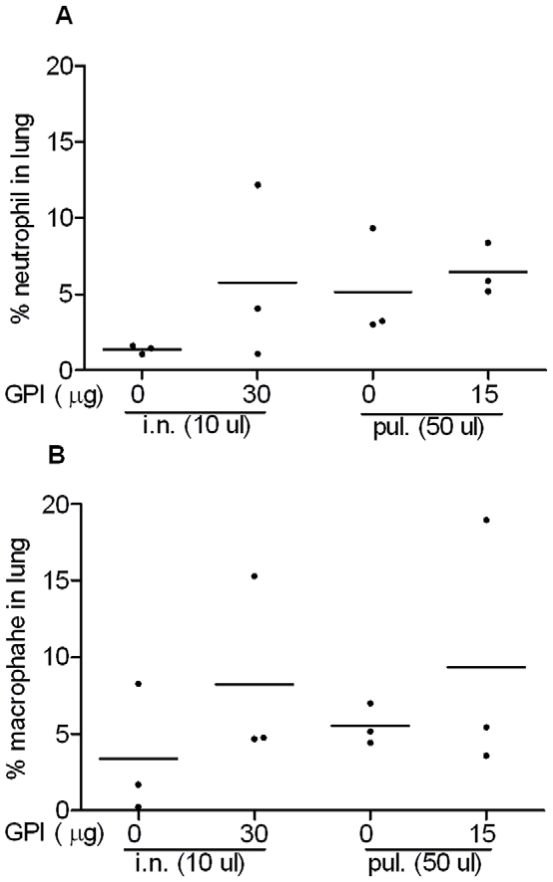
Effect of GPI-0100 on recruitment of inflammatory cells to the lung. Quantification of (A) neutrophils and (B) macrophages on micrographs as described in the legend to Figure 6. For each staining section, regions of interest (ROI) were selected using HistoQuest software. The total number of cells within the regions were defined by the nucleus staining. Neutrophils or macrophages are defined as nucleated brown cells. The results are presented as % neutrophils or % macrophages per stained sample from individual mouse.

In order to evaluate the safety of mucosal delivery of GPI-0100, mice received HBS or GPI-0100 alone via the intranasal or the intrapulmonary route on day 0 and 20. Mice were sacrificed 72 hr after the second administration. Lungs were collected for histopathological analysis of inflammatory cells. Histology images show that the lung structure was intact without severe cell infiltration in all experimental mice (Figure 6). HistoQuest counting of the images revealed that mice receiving GPI-0100 via the intranasal route had a higher number of neutrophils and macrophages in the lungs than the HBS-treated control mice, but the differences were not statistically significant (Figure 7). Pulmonary administration of HBS alone resulted in higher numbers of neutrophils and macrophages in the lungs when compared to intranasal HBS. However, GPI-0100 did not enhance the mild irritation caused by the delivery method itself. Thus, for the amounts investigated, mucosal delivery of GPI-0100 had only minor effects on lung histology and appeared to be safe.

## Discussion

In the present study we evaluated the immunogenicity and protective efficacy of a GPI-0100-adjuvanted A/PR/8 influenza subunit vaccine delivered via the intranasal or intrapulmonary route in a murine model system. The challenge experiment showed that GPI-0100-adjuvanted mucosal influenza vaccine could provide complete lung protection against influenza virus infection in contrast to non-adjuvanted mucosal vaccine. The strong lung protection was associated with strong serum IgG titers. Remarkably, GPI-0100 enhanced the serum IgG titers elicited by the intranasal and the intrapulmonary influenza vaccine up to 410 and 2700 times, respectively. In addition, GPI-0100 significantly boosted influenza-specific mucosal antibody responses elicited by the mucosal vaccines. The enhanced systemic and mucosal antibody responses were associated with enhanced influenza-specific IL-4 secreting T cell responses. These results were reproducibly observed for the immunization and the immunization/challenge study described in this paper and could be repeated in subsequent independent follow-up studies.

Our current study demonstrates that mucosal delivery of GPI-0100-adjuvanted vaccine resulted in strong Th2 type immune responses (IgG1 and IL-4) but relatively poor induction of Th1-related IgG2a or IFN-γ. Earlier studies on mucosal immunity indicate that the microenvironment of the respiratory tract tends to induce a Th2-oriented local immune response.[29]–[32] Obviously, GPI-0100 is not capable of overcoming this default immune response phenotype of the respiratory tract. In contrast, with parenteral administration we observed earlier that GPI-0100 significantly enhanced both IgG1 and IgG2a antibody responses elicited by influenza subunit vaccine and led to a more balanced Th1/Th2 antibody response. [20] Similar to our studies, research on a Porphyromonas gingivalis vaccine has also pointed out an effect of the administration route on GPI-0100 adjuvant activity. [21] Upon subcutaneous administration, GPI-0100-adjuvanted HagB antigen elicited a more Th1-skewed antibody response than the antigen alone. Intranasal administration of GPI-0100 adjuvanted HagB antigen, however, stimulated a robust IgG1 but a poor IgG2a response and resulted in a Th2-skewed antibody phenotype.

We furthermore observed a different immune-inducing potential of the two mucosal immunization routes investigated. Plain influenza vaccine was effective in inducing mucosal antibody responses when delivered via the intrapulmonary but not the intranasal route. In addition, with the same dose of GPI-0100 adjuvant, intrapulmonary vaccine elicited significantly higher mucosal and systemic antibody responses than intranasal vaccine. The difference in immunogenicity of mucosal vaccines delivered to different sites of the respiratory tract was also observed in several other vaccination studies, all pointing out that a good mucosal immunogenicity and induction of protective response were achieved by antigen delivery to the total respiratory tract (TRT), or the lower respiratory tract (LRT), but not the upper respiratory tract (UTR).[33]–[35] Studies on antigen deposition have shown that intranasal delivery of a large antigen volume results in antigen deposition in deeper locations along the respiratory tract while small volumes retain the antigen in the nose. [33], [36] Since we were interested in elucidating in how far the site of delivery is important for the adjuvant function of GPI-0100 we took care to ensure delivery to the nasal mucosa by using a small inoculum of 5 µl and to circumvent the nasal mucosa and deliver the vaccine exclusively to the lower respiratory tract by administering an aerosol to intubated mice. This allowed us to detect the particularly strong adjuvant function of GPI-0100 in the lungs.

With strict URT and LRT targeting, our data show that 15 µg GPI-0100 was sufficient for both intranasal and intrapulmonary influenza vaccine containing 1 µg HA to elicit substantial serum antibody responses after a single immunization. Another saponin-derived adjuvant that can easily be mixed with protein antigens and has been studied in the context of mucosal immunization against influenza is ISCOMATRIX™ (IMX). [35], [37]–[39] In a study of Coulter et al, IMX-adjuvanted split influenza vaccine delivered in a small volume of 12 µl induced comparable antibody titers as in our study. Yet, the amount of IMX used was with 100 µg much higher than the amount of GPI-0100 (15 µg) used here. Another study on IMX adjuvanted influenza vaccine (6.8 µg HA with 10 µg IMX) showed that a single dose immunization via the UTR failed to induce detectable serum antibody responses and lung protection in the immunized mice. [35] According to that study, an adjuvant effect of this low dose of IMX was only observed when the vaccine was delivered to the TRT while GPI-0100 clearly showed adjuvant activity in the UTR at a comparable dose. Thus, GPI-0100 is at least as potent as or even more potent than IMX as adjuvant for mucosal immunization via the respiratory tract.

Cholera toxin subunit B (CTB) is one of the most potent mucosal adjuvants and is the only mucosal adjuvant that has been incorporated into currently licensed mucosal vaccines. [40] Studies on CTB-adjuvanted mucosal influenza vaccine have shown that nasal IgA responses elicited by intranasal influenza vaccine containing 1 µg HA were boosted upon CTB adjuvantation up to 4- and 6-fold, with a 2- and 3-dose regimen respectively.[41]–[42] In addition, CTB enhanced systemic IgG responses elicited by the intranasal influenza vaccine for more than 4- and 250-fold, for the two regimens respectively. Here we observed that GPI-0100 mucosal adjuvantation enhanced nasal IgA by a factor of >2.5 and systemic IgG responses by a factor of 410 with a 2-dose regiment. For pulmonary immunization GPI-0100 enhanced nasal and pulmonary IgA by a factor of 7 and 110, respectively. Systemic IgG titers after two immunizations were enhanced by more than 2700-folds. Thus, GPI-0100 showed similar or even better mucosal adjuvant activity than the most potent adjuvant known to date.

Respiratory tract immunization has been shown to induce a heterogenous population of lymphocytes expressing different surface adhesion molecules. [2], [43] Depending on the expression profile of the surface markers, the activated lymphocytes can home to the mucosal priming site or to systemic lymphoid organs and blood later on. The capacity of respiratory tract immunization to integrate mucosal and systemic immunity is also shown in the present study with the induction of mucosal and systemic antibody responses. Upon influenza infection most probably both mucosal antibodies as well as systemic antibodies contribute to protection. [27], [28], [44], [45] In our study in which we used TRT virus challenge we observed a particularly high inverse correlation between serum IgG titers and virus titers detected in the lung supernatant of the immunized and challenged mice (Spearman r (coefficient) =  −0.69, p<0.0001). However, in natural infection IgA in the URT might be of high importance to prevent initial infection. In this sense, GPI-0100 adjuvanted subunit vaccine which can elicit both mucosal immune responses at the port of entry of influenza virus and systemic immune responses with proven significance for preventing LRT complications is ideal.

The safety of GPI-0100 mucosal administration was evaluated in the current study by lung histology analysis. Neutrophil and macrophage staining in the lung sections showed that GPI-0100 did not induce more severe inflammation than the mechanical irritation caused by intrapulmonary delivery itself. The latter was also observed in an earlier study. [46] To our knowledge, we are the first to show that mucosal delivery of a saponin-derived adjuvant to the nose or directly into the lungs is well tolerated.

In conclusion, our data show that GPI-0100 is a potent and well-tolerated mucosal adjuvant for influenza subunit vaccines. In the murine model system, GPI-0100 enhanced mucosal antibody responses in the respiratory tract elicited by both intranasal and intrapulmonary vaccines. Such mucosal antibodies can provide early neutralization before the attachment of influenza virus to the host cells. In addition, GPI-0100 adjuvanted mucosal vaccine induced strong systemic antibody responses, known to offer effective lung protection upon virus challenge. [27], [28] Furthermore, we showed that GPI-0100-adjuvanted influenza vaccine delivered via the intrapulmonary route was very potent and a much lower antigen dose could possibly be applied. Since aerosol inhalation devices for deep lung targeting in humans are available, intrapulmonary immunization offers an easy and promising strategy for future mucosal influenza vaccination.
